# COVID-19 Infection Stigma Scale: psychometric properties

**DOI:** 10.1186/s41983-021-00317-0

**Published:** 2021-05-17

**Authors:** Hayam Mohammed Elgohari, Medhat Mohamed Bassiony, Mohammad Gamal Sehlo, Usama Mahmoud Youssef, Heba Mohamed Ali, Islam Shahin, Dina Sameh Elrafey, Rehab Saeed Mahdy

**Affiliations:** 1 Department of Psychiatry, Faculty of Medicine, Zagazig University, Zagazig, Egypt; 2 Department of Psychology, Faculty of Arts, Zagazig University, Zagazig, Egypt; 3 Cairo Mood Center, Cairo, Egypt; 4 Department of Community, Environmental and occupational Medicine, Faculty of Medicine, Zagazig University, Zagazig, Egypt

**Keywords:** COVID-19, Stigma scale, Psychometric properties

## Abstract

**Background:**

Stigma has been noticed towards patients with COVID-19 in several regions of the world. This social discrimination has contributed to delay in diagnosis and treatment. Also, it may increase the suffering of the patients leading to poor outcome of the illness. Stigma can be assessed with the use of a valid and reliable instrument developed and adapted to our culture. Our objective was to analyze the psychometric properties of COVID-19 Infection Stigma Scale (CISS) for measuring the social stigma among patients with COVID-19 in Egypt. A cross-sectional study that included 182 COVID-19 patients was carried out. The reliability, the convergent validity, and the external and internal consistency of the scale were measured. Factor analysis was used to exclude the weak items.

**Results:**

The mean of the COVID-19 Infection Stigma Scale scores was 34.97±10.35 which was higher than 50% of the score. Absence of the floor and ceiling effects was observed. Cronbach’s alpha coefficient for scale reliability ranged from 0.75 to 0.94 with 0.82 for the total score. The convergent validity coefficients ranged from 0.36 to 0.63. Test-retest validity Pearson’s correlation coefficients ranged from 0.72 to 0.92 with 0.89 for the total score. The split half correlation coefficient was 0.86, and the reliability coefficient was 0.92. Both were acceptable correlation coefficients for internal consistency of the scale. Factor analysis showed two factors had latent root greater than 1. The rotated component matrix of the 2 factors revealed that all questions had *r* value more than 0.30, which means that no need to exclude any of them.

**Conclusion:**

The results showed that the COVID-19 Infection Stigma Scale is a valid and reliable instrument for the Egyptian people.

**Supplementary Information:**

The online version contains supplementary material available at 10.1186/s41983-021-00317-0.

## Background

A stigma is a standardized disgrace image that is held by a community toward certain people [1]. People stigmatize others who present a threat to effective group function [2]. In case of infectious disease stigmas, the disgrace is being infected with a contagious disease. Infectious diseases threaten a community’s ability either by limiting infected persons to perform their roles within a society or by killing them. In addition, infectious diseases by spreading from member to member through interaction are extremely affected by the social nature of groups [3].

Stigmatization may increase the consequences of a disease in many ways. First, stigmatization may increase the suffering of patients. Second, patients may delay or avoid seeking medical advice, making the disease control difficult by public health authorities. Third, professionals and volunteers working in the field may also become stigmatized, leading to more stress and burnout [4]. Finally, stigmatization may generate considerable economic losses if people avoid groups or whole regions associated with the disease [5].

The COVID-19 pandemic is first identified in Wuhan, China, and now spread worldwide. It is transmitted primarily by respiratory droplets and close contact. People became frightened and concerned because of lack of understanding and uncertainty [6, 7]. When the outbreak is caused by a new virus, rumors and false information rapidly spread. Stereotypes rapidly appear towards persons who have or may have the illness. In the USA and Europe, for example, Asian people have been treated with suspicion and blamed for COVID-19. Also, some people worry that individuals who have recently completed quarantine have COVID-19 and are contagious, even if there is no current evidence to suggest that case. Blaming the infected people in this manner is hurtful and dangerous, and it may lead to misplaced anger, hostility, or even suicide. It also creates hardships that obstruct the response to the pandemic [8].

To our knowledge, there is no tool to asses COVID-19 stigma worldwide. The aim of this study was to test the validity and reliability of the predesigned scale for measuring stigma associated with the COVID-19 infection.

## Methods

### Study design and place of study

A cross-sectional study was carried out in a governorate in Egypt. In the governorate, COVID-19 patients’ management was restricted to isolated governmental hospitals. The patients received the scale either through a paper form or through an electronic Google form sent to them especially in the re-test application.

### Population and sample

The study sample included all positive COVID-19 cases diagnosed by polymerase chain reaction (PCR), which equal to 1399 cases from beginning of the pandemic till 15th June 2020. The sample size was calculated through the Epi-Info (Epidemiological information package) software version 6.1 [9], according to the following collected data: Total number of COVID-19 cases in the governorate was 1399. The frequency of social stigma among infectious diseases in a previous study was found to be 16.1% [10]. So, at a confidence interval of 95%, the estimated sample size was calculated to be 182 cases. The following inclusion criteria were applied to COVID-19 patients of both sexes, older than 18 years and willing to participate. Patients with psychiatric disorders, substance use disorders, delirium, or with respiratory distress were excluded from the study.

### Development of the scale

To develop the COVID-19 Infection Stigma Scale, an extensive literature review was conducted to assess all general scales for stigma [11]. We considered the Stigma Scale for Chronic Illnesses 8-Item [12], the Reece Stigma Scale [13], and the Arabic Self–Stigma Scale [14]. This is a self-report quantitative tool that specifically measures the stigma related to COVID-19 infection. The Arabic scale was designed and revised by the authors.

It consists of 14 questions related to feelings such as fear, guilt, and sorrow in coping with the disease, and attitude and self-feeling towards the infection and also anxiety and fear of the reaction of others.

The items were graded on a four-point Likert scale (1 to 4): never (1), rarely (2), usually (3), and always (4). The item scores from the questionnaire were summed, and the scores ranged respectively from 14 to 56 with 14 indicating no stigma and 56 indicating the highest level of stigma.

### Pilot test

The pilot test was intended to collect data on the initial psychometric properties of the scale in Arabic version and permitted a simulation of the field study. The pilot test was applied on 10% of the sample size (18 patients); they were not included in the study population.

### Statistical analysis

The data were collected between April 2020 and July 2020. Double-data entry was used to avoid possible transcription errors. The software adopted was the Statistical Package for the Social Sciences (SPSS), version 25.0 [15]. The presence of the floor and ceiling effects was verified, and forms with typical answers were excluded from the research when present (the answers are concentrated in the lowest or highest scale score) because they negatively affect the responsiveness of the instrument. The reliability of the scale was measured using Cronbach’s alpha. The coefficients were estimated, considering results above 0.70 as acceptable [16]. To analyze the construct validity, the convergent validity of the scale was used, with the aim of verifying the correlations among items and total score [17]. The external consistency of the scale was analyzed by test-retest validity, the participants were asked to fill the scale two times with 2 weeks interval, and correlations between answers in the 1st and 2nd times were calculated [18]. Also, internal consistency of the scale was analyzed using split half reliability coefficient. Acceptable correlation coefficients for all mentioned methods should be more than 0.30 [19]. Kaiser-Meyer-Olkin measure of sampling adequacy was used to confirm adequacy of sample size (value of more than 0.50 means adequate sample size). Bartlett’s test of sphericity was used to confirm statistically significant relationship between items, and *p* value less than 0.05 is considered to be significant [20]. Factor analysis was applied to exclude any weak items in the scale. The scree plot graph and extraction of the component factor were used to find the factors with latent root greater than the correct one. Extraction method by principal component analysis and rotation method by Varimax with Kaiser normalization was used to find saturation of items in each factor. Items with saturation more than 0.30 was considered to be strong item [21]. The 50% of the total score (28) was consider to be the cutoff value to discriminate between cases who had stigma and cases who had not, where cases who had > 28 are considered to have stigma.

## Results

One hundred and eighty-two patients who were diagnosed with COVID-19 and confirmed with PCR test at hospitals of the governorate participated in the study. The patients’ age ranged between 18 and 66 years, with a mean age of 38.73 (SD 11). Hundred and five (57.7%) of the patients were females, 26 (14.3%) were unemployed, and 68 (37.4%) were health care workers (HCWs). One out of three and one out of four has either a university degree or post-graduate education respectively. The majority of the studied cases were married (70.3%), and 102 patients (56%) were from rural areas, as shown in Table 1.

**Table 1:** Sociodemographic characteristics of the studied group

Variable		(*n*=182)	
Age	Mean ± SD	38.73 ± 11	
	Range	18–66	
Variable		**No.**	**%**
Sex	Female	105	57.7
	Male	77	42.3
Occupation	Not working or housewife	26	14.3
	Retired	4	2.2
	Student	15	8.2
	Skilled and worker	20	11
	Free business	9	4.9
	Employer	13	7.1
	Specialist	27	14.8
	HCWs	68	37.4
Education	Read and write	7	3.8
	Middle	29	15.9
	High	37	20.3
	University	63	34.6
	Post-university	46	25.3
Residence	Rural	102	56
	Urban	80	44
Marital status	Single	40	22
	Married	128	70.3
	Divorced	5	2.7
	Widow	9	4.9

*SD* standard deviation

In Table 2, after excluding forms with typical answers (the answers were located in the lowest or highest scale score), the total patients’ number became 166. The means, medians, standard deviations, minima, and maxima of the COVID-19 Infection Stigma Scale scores were calculated (34.97, 38, 10.35, 14, and 56 respectively). As observed, the mean scores were higher than 50%. As for the floor and ceiling effects, the absence of these effects in the dimensions was observed, that is, no answers higher than 15% were found at the top and bottom of the scale, which favors its responsiveness.

**Table 2:** Measures of the scale and floor and ceiling effect of answers in the scales

Variable		(*n*=182)	
Total score	Mean ± SD	34.97 ± 10.53	
	Median	38	
	Range	14–56	
Variable		**No.**	**%**
Floor	Answers in the minimum score	6	3.3
Ceiling	Answers in the maximum score	3	1.7

*SD* standard deviation

The reliability was analyzed and determined using Cronbach’s alpha coefficient. The coefficients if excluded the item and the total alpha of the scale dimensions are displayed in Table 3. When testing the impact of removing each item on the total alpha coefficient of the scale dimensions, changes were observed, ranging from 0.75 (I am afraid that my family will stay away from me because of my infection) to 0.94 (I feel that it is a must to hide the news of my infection from others) and 0.82 for the total score. Cronbach’s alpha was considered good because all items had alpha more than 0.70.

**Table 3:** Alpha coefficients if excluding the item and total alpha of the dimensions

Variable	Cronbach’s alpha
1. I feel shy and ashamed of my infection with coronavirus.	0.86
2. I am frustrated and disappointed because of my infection with coronavirus.	0.85
3. I feel like an unwanted person.	0.88
4. I feel rejected by others because of my illness	0.83
5. I feel that being infected with coronavirus is a punishment for something I did in the past.	0.77
6. Having corona makes me feel inferior and less than others.	0.92
7. Having corona harms the reputation of my family.	0.80
8. I am afraid of being refused and not accepted by my co-workers.	0.90
9. I will be isolated and away from others to avoid being mistreated.	0.82
10. I am afraid that I will lose my work because of my infection.	0.91
11. I am afraid that my family will stay away from me because of my infection.	0.75
12. I am afraid of being avoided by neighbors in the house because of my infection.	0.82
13. I was late in seeking treatment for fear that someone would know that I had corona virus infection.	0.85
14. I feel that it is a must to hide the news of my infection from others.	0.94
Total	0.82

Regarding split half reliability coefficient of the scale, correlation coefficient was 0.86, and reliability coefficient was 0.92; both are considered acceptable correlation coefficients for internal consistency of the scale.

The construct validity was verified using the convergent validity of the scale; Pearson’s correlation coefficients were used between the items and the total score of the scale. The convergent validity coefficients are displayed in Table 4. It ranged from 0.36 (having corona harms the reputation of my family) to 0.63 (I am afraid of being refused and not accepted by my co-workers). All the studied items obtained coefficients >0.30 which are acceptable correlation coefficients.

**Table 4:** Pearson’s correlation coefficients of each item of stigma scale with total score and between participant answers in the 1st and 2nd time

Variable	*r*1	*P*	*r*2	*P*
1. I feel shy and ashamed of my infection with coronavirus	0.47	<0.001**	0.86	<0.001**
2. I am frustrated and disappointed because of my infection in coronavirus.	0.58	<0.001**	0.89	<0.001**
3. I feel like an unwanted person.	0.40	0.004**	0.80	<0.001**
4. I feel rejected by others because of my illness	0.46	<0.001*	0.83	<0.001**
5. I feel that being infected with coronavirus is a punishment for something I did in the past.	0.39	0.006*	0.74	<0.001**
6. Having corona makes me feel inferior and less than others.	0.50	<0.001**	0.91	<0.001**
7. Having corona harms the reputation of my family	0.36	0.01*	0.72	<0.001**
8. I am afraid of being refused and not accepted by my co-workers.	0.63	<0.001**	0.89	<0.001**
9. I will be isolated and away from others to avoid being mistreated	0.52	<0.001**	0.73	<0.001**
10. I am afraid that I will lose my work because of my infection.	0.47	<0.001**	0.79	<0.001**
11. I am afraid that my family will stay away from me because of my infection.	0.49	<0.001**	0.81	<0.001**
12. I am afraid of being avoided by my neighbors in the house because of my infection.	0.52	<0.001**	0.88	<0.001**
13. I was late in seeking treatment for fear that someone would know that I had corona.	0.48	<0.001**	0.85	<0.001**
14. I feel that it is a must to hide the news of my infection from others.	0.62	<0.001**	0.92	<0.001**
Total	–	–	0.89	<0.001**

*r1* Pearson’s correlation coefficient of each item of Stigma scale with total score, *r2* Pearson’s correlation coefficients between participant answers in the 1st and 2nd time

*Significant (*P*<0.05), **highly significant (*P*<0.001)

The external consistency of the scale was analyzed by test-retest validity. Pearson’s correlation coefficients were used between the items in the 1st and 2nd time of patients’ answers. The results are displayed in Table 4. It ranged from 0.72 (having corona harms the reputation of my family) to 0.92 (having corona makes me feel inferior and less than others) and 0.89 for the total score. All the studied items obtained coefficients >0.30 which are acceptable correlation coefficients.

Adequate sample size was tested by using Kaiser-Meyer-Olkin measure of sampling adequacy which was 0.94. Also, Bartlett’s test of sphericity was 0.98, and *p* value <0.001 indicate highly statistical significant relationship between items.

**Fig. 1:**
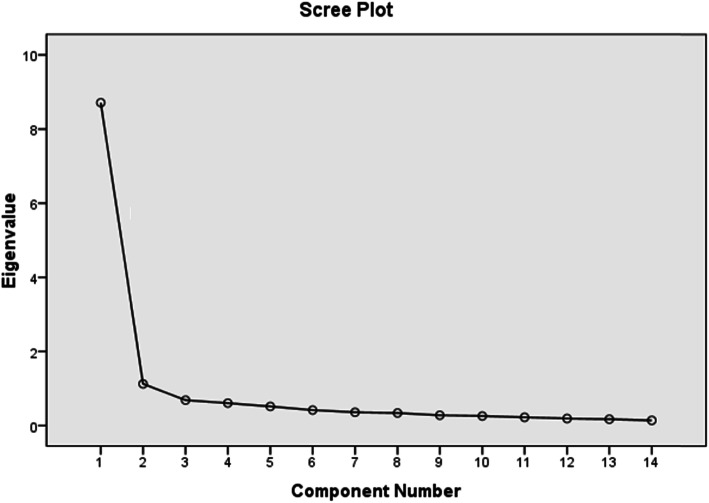
Scree plot showing the result of extraction of the component factor

Table 5 and the scree plot graph (Fig. 1) show the result of extraction of the component factor using the components principal on commonality transactions for the variable; we have arrived at two factors that had latent root greater than 1. The percentages of interpretation of variances from the total variance of each factor were reached, where the 1st factor has the potential root equal to 8.71 and explains 62.21% of the total variance. On the other hand, the 2nd factor has the potential root equal to 1.13 and explains 70.26% of the total variance.

**Table 5:** Result of extraction of the component factor and rotated component matrix

Variable		Component	
		1	2
**Initial eigenvalues**	**Total**	8.710	1.126
	**% of variance**	62.213	8.044
	**Cumulative %**	62.213	70.257
Question	q1	0.399	**0.785**
	q2	0.537	**0.661**
	q3	**0.645**	0.524
	q4	**0.796**	0.352
	q5	0.445	**0.610**
	q6	**0.592**	0.532
	q7	**0.816**	0.360
	q8	0.313	**0.791**
	q9	**0.749**	0.265
	q10	0.241	**0.791**
	q11	0.454	**0.568**
	q12	**0.855**	0.317
	q13	0.249	**0.824**
	q14	**0.809**	0.310

Extraction method: principal component analysis. Rotation method: Varimax with Kaiser normalization

Table 5 showed the results of rotated component matrix on the 2 factors which revealed that the 1st factor explains 62.21% and included question numbers 3, 4, 6, 7, 9, 12, and 14, while the 2nd factor explains 70.26% and included questions 1, 2, 5, 8, 10, 11, and 13. All questions had *r* values more than 0.30 which means that there is no need to exclude any of them.

## Discussion

The objective of the study was to test the psychometric properties of the COVID-19 Infection Stigma Scale among an Egyptian sample of COVID-19 patients. It is a novel tool that aimed to assess and measure the COVID-19-related stigma. It will be of great benefit for further research, application in other cultures, and for decreasing the negative consequences of stigma on the patients. The scale is clear and concise and easy to be applied. The four-point Likert scale gave the chance for wide range of responses. It covers the internal aspect of stigmatization which represent the self-feeling (items from 1 to 6) and the external aspect that appears within the treatment of the others for example at work or at neighborhood (items 7 to 14). The scale was considered to be a very good tool for measuring stigma because of the absence of the floor and ceiling effects, very good reliability (Cronbach alpha range 0.75–0.94 and 0.82 for the total score), strong convergent validity (*r* range 0.36–0.63), strong external consistency (test-retest *r* range 0.72 to 0.92 and 0.89 for the total score), and strong internal consistency (split half correlation *r* was 0.86 and reliability *r* was 0.92). Further verification, such as using confirmatory factor analysis, was a strength for the scale where all questions had *r* values more than 0.30 which means that there is no need to exclude any of them. The limitation of this study is the selection of the patient sample from one governorate in Egypt. In addition, the questions in the scale did not include reverse-coded questions.

## Conclusion

The scale showed favorable initial psychometric properties in the pilot test, which sustains the validity and reliability of the scale. It is the first scale validated in the Arabic language, which might be helpful for other Arabic-speaking countries that are liable to COVID-19 stigma. The COVID-19 Infection Stigma Scale can stimulate the advancement of operational research and the development of strategies to reduce the stigma related to COVID-19. It could be used in other cultures after translation into other languages. We recommend further studies to assess the risk factors of stigma in our society.

## Supplementary Information

**Additional file 1.**

## Data Availability

All data generated or analyzed during this study are included in this published article.
