# Supplementary material for: COVID-19 Infection Stigma Scale: psychometric properties
Source: Egypt J Neurol Psychiatr Neurosurg. 2021 May 17;57(1):61. doi: 10.1186/s41983-021-00317-0 (PMC8127429; doi:10.1186/s41983-021-00317-0)
Supplement: Supplementary file 1 — Additional file 1. [file 41983_2021_317_MOESM1_ESM.docx]

**Additional informations**

**Appendix:**

**The English version of COVID-19 Infection Stigma Scale (CISS)**

The items were graded on a four-point Likert scale (1 to 4), Never (1) , Rarely (2), Usually (3) and Always (4).

Please respond to each item according to how you feel

|  |  | **Always** | **Usually** | **Rarely** | **Never** |
| --- | --- | --- | --- | --- | --- |
| **1.** | I feel shy and ashamed of my infection with Corona virus. |  |  |  |  |
| **2.** | I am frustrated and disappointed because of my infection with Corona virus. |  |  |  |  |
| **3.** | I feel like an unwanted person. |  |  |  |  |
| **4.** | I feel rejected by others because of my illness. |  |  |  |  |
| **5.** | I feel that being infected with Corona virus is a punishment for something I did in the past. |  |  |  |  |
| **6.** | Having Corona makes me feel inferior and less than others. |  |  |  |  |
| **7.** | Having Corona harms the reputation of my family. |  |  |  |  |
| **8.** | I am afraid of being refused and not accepted by my co-workers. |  |  |  |  |
| **9.** | I will be isolated and away from others to avoid being mistreated. |  |  |  |  |
| **10** | I am afraid that I will lose my work because of my infection. |  |  |  |  |
| **11.** | I am afraid that my family will stay away from me because of my infection. |  |  |  |  |
| **12.** | I am afraid of being avoided by neighbors in the house because of my infection. |  |  |  |  |
| **13.** | I was late in seeking treatment for fear that someone would know that I had a Corona virus infection. |  |  |  |  |
| **14.** | I feel that it is a must to hide the news of my infection from others. |  |  |  |  |

- **النسخه العربية لمقياس وصمة العدوى بالكوفيد 19**

**التعليمات:**

**من فضلك:**

**- أمامك عدد من العبارات تصف ما تشعر به بعد الإصابة بفيروس كورونا**

**والمطلوب أن تقرأ كل عبارة بعناية ثم اختر استجابة واحدة على كل عبارة من بين الاستجابات التالية:-**

**دائماً**: إذا كانت العبارة تصف مشاعرك بشكل دائم.

**غالباً**: إذا كانت العبارة تصف مشاعرك في أغلب الأوقات.

**نادراً**: إذا كانت العبارة نادراً أو قليلاً ما تصف مشاعرك.

**أبداً**: إذا كانت العبارة لا تنطبق عليك.

مفتاح تصحيح المقياس : **دائماً = 4 – غالباً = 3 – نادرا=2 – أبدا=1**

| **م** | **العباره** | **دائما** | **غالبا** | **نادرا** | **ابدا** |
| --- | --- | --- | --- | --- | --- |
| **1** | أشعر بالخجل والخزي لإصابتي بفيروس كورونا. |  |  |  |  |
| **2** | أشعر بالإحباط وخيبة الأمل بسبب إصابتي بكورونا . |  |  |  |  |
| **3** | . أشعر بأنني شخص غير مرغوب فيه. |  |  |  |  |
| **4** | أشعر بالرفض من الآخرين بسبب مرضي بكورونا. |  |  |  |  |
| **5** | أشعر أن إصابتي بفيروس كورونا عقاب على شيء فعلت بالماضي. |  |  |  |  |
| **6** | إصابتي بكورونا تجعلني أشعر بالنقص وأنني أقل من الآخرين. |  |  |  |  |
| **7** | إصابتي بكورونا يسيء إلى سمعة أفراد أسرتي. |  |  |  |  |
| **8** | أخاف من رفض زملاء العمل وعدم تقبلهم لي. |  |  |  |  |
| **9** | سأكون منعزلا وبعيدا عن الآخربن لتجنب سوء معاملتهم. |  |  |  |  |
| **10** | أخشى أن أفقد وظيفتي (عملي) بسبب إصابتي بكورونا. |  |  |  |  |
| **11** | أخاف أن يبتعد عني أفراد أسرتي بسبب إصابتي بكورونا. |  |  |  |  |
| **12** | أخشى أن يقاطعني جيراني في السكن (المنزل) بسبب إصابتي بفيروس كورونا. |  |  |  |  |
| **13** | تأخرت في طلب العلاج خوفا" من أن يعرف أحد بإصابتي بكورونا. |  |  |  |  |
| **14** | أشعر بضرورة إخفاء خبر إصابتي بفيروس كورونا عن الآخرين. |  |  |  |  |
